# The factors driving self-efficacy in intractable chronic pain patients: a retrospective study

**DOI:** 10.1186/s13018-019-1535-9

**Published:** 2019-12-30

**Authors:** Hironori Tsuji, Tomoko Tetsunaga, Tomonori Tetsunaga, Keiichiro Nishida, Haruo Misawa, Toshifumi Ozaki

**Affiliations:** 1 Department of Orthopaedic Surgery, Okayama University Graduate School of Medicine, Dentistry, and Pharmaceutical Sciences, 2-5-1 Shikata-cho, Kitaku, Okayama, Okayama 700-8558 Japan; 2 Department of Orthopaedic Surgery, Okayama University Hospital, 2-5-1, Shikata-cho, Kitaku, Okayama, Okayama 700-8558 Japan

**Keywords:** Self-efficacy, Chronic pain, Fear-avoidance model

## Abstract

**Background:**

The fear-avoidance model is a theoretical paradigm for explaining acute and chronic pain. In this model, pain catastrophizing plays an important role. On the other hand, self-efficacy influences whether patients view their pain optimistically, ultimately preventing the conversion of pain into intractable pain. The aim of the present study was to evaluate the factors that influence self-efficacy in patients with chronic pain.

**Methods:**

Study participants included 147 outpatients (35 men, 112 women) with intractable chronic pain who visited our hospital between September 2014 and July 2015. Their mean age was 71.0 (range 32–92) years. Pain sites were as follows: low back, 97 patients; knee, 71 patients; shoulder, 34 patients; and hip, 15 patients. All patients were assessed using the following measures: Numeric Rating Scale (NRS), Pain Catastrophizing Scale (PCS), Hospital Anxiety and Depression Scale (HADS), Pain Disability Assessment Scale (PDAS), and Pain Self-Efficacy Questionnaire (PSEQ). All participants were further divided into two groups based on median PSEQ scores (group L: PSEQ of 35 points or less, *n* = 74; group H: PSEQ greater than 35 points, *n* = 73). The factors that influenced self-efficacy in these patients were analyzed using univariate and multiple linear regression analyses.

**Results:**

Significant differences were observed in gender; pain duration; and NRS, PDAS, HADS, and PCS scores between group L and group H. Multiple linear regression analysis revealed that self-efficacy was correlated with PDAS score, HADS depression score, and pain duration.

**Conclusions:**

Patients with longer pain duration indicated greater self-efficacy and patients with higher pain disability and depression exhibited lower self-efficacy.

## Background

**Fig. 1:**
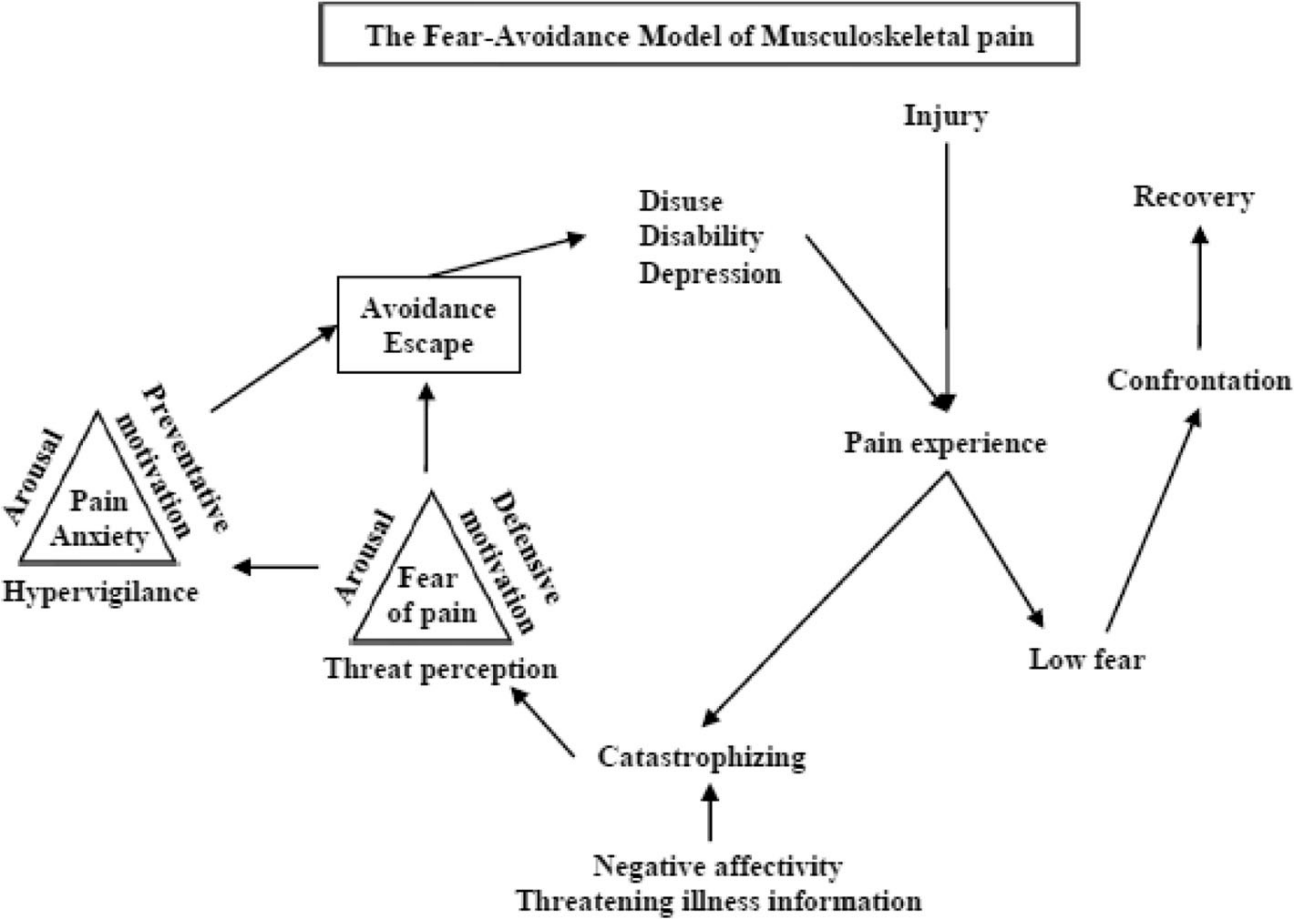
The fear-avoidance model for musculoskeletal pain. This figure is reprinted after obtaining permission from Springer Nature Customer Service Centre GmbH: Springer Nature [11], copyright 2007

It is widely known that chronic pain results in substantial medical expenses and economic loss due to prolonged temporary employment leaves [1–4]. The reported worldwide prevalence of chronic pain ranges from 13.5 to 47%, reflecting a large number of individuals who experience chronic pain at any given time [1, 5, 6]. Pain chronicity has implications for the physical and psychological functioning of patients, with their treatment often complicated by psychological comorbidities including anxiety and depression [7–9]. Vlaeyen and Linton proposed a cognitive behavioral model of chronic pain that has been termed the fear-avoidance model [10]. A version modified slightly by Asmundson et al. further includes fear and anxiety, with pain catastrophizing contributing to misinterpretation and ultimately a vicious chronic pain cycle, which convert the pain into intractable pain (Fig. 1) [11, 12]. When pain is perceived as non-threatening, patients are likely to set confrontation with pain and maintain engagement in daily activities. As this confrontation with pain, we consider that self-efficacy serves as a protective factor against the development of a vicious chronic pain cycle.

Self-efficacy is defined as confidence that a patient can successfully execute a course of action to produce a desired outcome [13]. Given this definition, self-efficacy may also determine how much effort and persistence individuals exhibit in the face of difficulties, such as chronic pain, which may allow individuals with high levels of self-efficacy to address their pain more directly via pain control or management strategies [13, 14]. Self-efficacy also allows individuals to interpret their pain more optimistically, thus mediating the relationship between pain and disability in individuals with chronic musculoskeletal pain. High self-efficacy is ultimately associated with lower levels of reported pain intensity, disability, and better physical functioning [15]. However, the factors which affect self-efficacy in patients with chronic pain remain unknown. Therefore, the objective of the present retrospective study was to assess the factors that mediate self-efficacy in patients with intractable chronic pain.

## Methods

### Participants

The present retrospective study was performed at the author’s institution. Ethical approval was obtained from the hospital board of ethics. Participants included 147 outpatients (35 males, 112 females) with intractable chronic pain who visited our pain liaison outpatient clinic between September 2014 and July 2015. Inclusion required pain persisting longer than 3 months without relief after conservative treatment (rest, use of a brace, pain medication, and physical therapy). The exclusion criteria included ongoing litigation, dementia, delirium, or other conditions that made completing questionnaires difficult.

### Pain assessment

The Numeric Rating Scale (NRS) for pain is a reliable, valid, and widely used tool for the self-evaluation of chronic pain intensity [16]. NRS scores range from 0 to 10, with 0 representing no pain and 10 representing the worst imaginable pain.

### Assessment of self-efficacy

The Pain Self-Efficacy Questionnaire (PSEQ) score was used to assess participant self-efficacy (Table 1). This is a Likert-type 10-item questionnaire composed of items which are scored from 0 (not at all confident) to 6 (completely confident), with total scores ranging from 0 to 60. A higher score indicates greater self-efficacy and functioning despite ongoing pain [17]. We used the PSEQ-J (Japanese), which has adequate psychometric properties to support its use in both clinical and research settings and is particularly associated with social activity [18].

**Table 1:** Pain Self-Efficacy Questionnaire

1.	I can enjoy things, despite the pain.
2.	I can do most of the household chores (e.g., tidying-up, washing dishes, etc.), despite the pain.
3.	I can socialize with my friends or family members as often as I used to do, despite the pain.
4.	I can cope with my pain in most situations.
5.	I can do some form of work, despite the pain (“work” includes housework, paid and unpaid work).
6.	I can still do many of the things I enjoy doing, such as hobbies or leisure activity, despite pain.
7.	I can cope with my pain without medication.
8.	I can still accomplish most of my goals in life, despite the pain.
9.	I can live a normal lifestyle, despite the pain.
10.	I can gradually become more active, despite the pain.

Each of the items is scored on a 7-point scale (0 = not at all confident, 6 = completely confident). The total score can range from 0 to 60 points

### Assessment of pain catastrophizing

The Pain Catastrophizing Scale (PCS), which is used to measure pain catastrophizing, is a 13-item questionnaire composed of items assessing rumination, magnification, and helplessness [19]. Rumination (items 8–11) refers to “the fact that the patient cannot get the idea of pain out of his/her head and cannot stop thinking about the pain,” while magnification (items 6, 7, and 13) refers to “the exaggeration of the threatening properties of the painful stimulus,” and helplessness (items 1–5 and 12) refers to “the estimation that the person is unable to do anything to influence the pain.” The PCS is scored on a scale from 0 to 52, with each item rated on a 5-point scale (0: not at all to 4: all the time). A higher score indicates a greater degree of pain catastrophizing.

### Assessment of anxiety and depression

The Hospital Anxiety and Depression Scale (HADS) was used to assess anxiety and depression [20, 21]. The HADS is composed of a 7-item depression scale and a 7-item anxiety scale, with each item scored from 0 to 3 and scores ranging from 0 to 21. A higher score indicates the presence of depression and/or anxiety.

### Assessment of physical disability

The Pain Disability Assessment Scale (PDAS) was used to assess the degree to which intractable chronic pain interfered with various activities of daily living during the previous week [22]. The PDAS is composed of 20 items, each rated on a 4-point scale (from 0: pain did not interfere with this activity to 3: pain interfered with this activity). The PDAS scores range from 0 to 60, with a higher score indicating greater pain-related interference.

### Statistical analyses

All patients were divided into two groups based on a median PSEQ score of 35 points. Patients with PSEQ scores of 35 points or lesser were included in the low PSEQ group (group L), and patients with scores greater than 35 points were included in the high PSEQ group (group H). Patients’ background and measured factors were compared between the two groups using chi-square and Mann-Whitney *U* tests and subjected to univariate analyses. Then, factors that influenced the PSEQ score in all patients as well as female patients were identified using multivariate linear regression analyses. Potential predictive variables were included in the multivariate model when *p* < 0.05 was obtained via univariate analyses. A multiple regression model and 95% confidence intervals were used to identify the factors that significantly influenced PSEQ scores. Statistical analyses were conducted using SPSS version 23.0 (IBM Corporation, Armonk, NY, USA) for Windows (Microsoft Corporation, Redmond, WA, USA).

## Results

### Participants

In the present study, 147 patients with intractable chronic pain who visited our pain liaison outpatient clinic and completed all questionnaires were included. Pain sites included the low back (*n* = 97), knee (*n* = 71), shoulder (*n* = 34), and hip (*n* = 15). Patients with two or more pain sites were also included. The mean participant age was 71.0 years (range 32–92 years), with a mean duration from pain onset to study enrollment of 58.0 months (range 3–624 months). Table 2 shows the background and measured variables for all participants by gender.

**Table 2:** Patients’ background and measured parameters by gender

	Total (*n* = 147)	Male (*n* = 35)	Female (*n* = 112)	*p* value
Age (years)	71.2 ± 12.2	69.9 ± 12.7	71.6 ± 12.8	0.11
Pain duration (months)	58.0 ± 92.7	44.1 ± 71.7	62.3 ± 98.2	0.39
PSEQ (points)	35.0 ± 14.2	36.6 ± 13.4	30.0 ± 15.6	0.03*
NRS (points)	5.2 ± 1.9	5.7 ± 2.1	5.1 ± 1.8	0.16
PCS (points)	26.4 ± 11.3	27.9 ± 10.3	25.9 ± 11.6	0.46
HADS anxiety (points)	3.9 ± 3.3	4.2 ± 2.9	3.9 ± 3.4	0.29
HADS depression (points)	4.7 ± 3.5	5.3 ± 3.5	4.4 ± 3.5	0.14
PDAS (points)	20.6 ± 13.0	23.8 ± 15.7	19.6 ± 11.9	0.19
Pain site (number of patients)				
Low back	97 (66.0%)	27 (77.1%)	70 (62.5%)	0.15
Knee	71 (48.3%)	13 (51.8%)	58 (37.1%)	0.13
Shoulder	34 (23.1%)	6 (17.1%)	28 (25.0%)	0.33
Hip	15 (10.2%)	2 (5.7%)	13 (11.6%)	0.31

Data are expressed as mean ± standard deviation or number (percentage)

*PSEQ* Pain Self-Efficacy Questionnaire, *NRS* Numeric Rating Scale, *PCS* Pain Catastrophizing Scale, *HADS* Hospital Anxiety and Depression Scale, *PDAS* Pain Disability Assessment Scale

Asterisks show statistical significance, *p < 0.05

### Factors affecting self-efficacy

All patients were divided into two groups in accordance with the median PSEQ scores of 35 points. Group L (< 35 points) included 74 patients, and group H (≥ 35 points) included 73 patients. Chi-square test for binary variables and Mann-Whitney *U* test for continuous variables were performed to compare the two groups. Table 3 shows the demographics of both groups. No significant difference was found between the two groups in age and pain site (*p* = 0.32 and 0.34, respectively). Significant differences were found between the two groups in gender, pain duration, NRS, PCS, HADS anxiety, HADS depression, and PDAS (*p* = 0.02, 0.00, 0.01, 0.00, 0.00, 0.00, and 0.00, respectively).

**Table 3:** Univariate analyses for patients’ background and measured variables

	Group L (*n* = 74)	Group H (*n* = 73)	*p* value
Age (years)	70.2 ± 13.4	72.2 ± 12.0	0.32
Gender (male/female)	24/50	12/61	0.02*
Pain duration (months)	36.7 ± 44.3	66.2 ± 80.5	0.00**
PSEQ (points)	23.2 ± 9.6	46.4 ± 6.2	0.00**
NRS (points)	5.6 ± 2.0	4.8 ± 1.8	0.01*
PCS (points)	29.8 ± 10.4	22.9 ± 11.2	0.00**
HADS anxiety (points)	4.8 ± 3.6	3.1 ± 2.6	0.00**
HADS depression (points)	6.1 ± 3.7	3.2 ± 2.4	0.00**
PDAS (points)	24.1 ± 13.9	17.0 ± 11.0	0.00**
Pain site (number of patients)			
Low back	54 (73.0%)	43 (58.9%)	0.07
Knee	30 (40.5%)	41 (56.2%)	0.06
Shoulder	19 (25.7%)	15 (20.5%)	0.46
Hip	8 (10.8%)	7 (9.6%)	0.80

Data are expressed as mean ± standard deviation or number (percentage)

*PSEQ* Pain Self-Efficacy Questionnaire, *NRS* Numeric Rating Scale, *PCS* Pain Catastrophizing Scale, *HADS* Hospital Anxiety and Depression Scale, *PDAS* Pain Disability Assessment Scale

Asterisks show the statistical significance, *p < 0.05, **p < 0.01

Next, multiple stepwise linear regression analyses were used to investigate the relationship between PSEQ scores (the response variable) and other variables. The explanatory variables included gender; pain duration; and NRS, PCS, HADS anxiety, HADS depression, and PDAS scores. We found that PSEQ scores (*y*) were positively correlated with pain duration (*x*_1_) and negatively correlated with HADS depression (*x*_2_) and PDAS (*x*_3_) scores for all patients (Table 4). These results yielded the following prediction formula: *y* = 46.74 + 0.02*x*_1_ − 1.07*x*_2_ − 0.39*x*_3_. The adjusted coefficient of determination was 0.29, and all *p* values were < 0.05; this indicated that the variables chosen for analysis had good explanatory power.

**Table 4:** Correlations between measured variables and PSEQ scores

Variable	Partial regression coefficient	Standard error	T-score	*p* value
HADS depression	− 1.07	0.31	− 3.42	0.00
PDAS	− 0.39	0.08	− 4.67	0.00
Pain duration	0.02	0.01	2.05	0.04
Constant term	46.74	2.14	21.87	0.00

*PSEQ* Pain Self-Efficacy Questionnaire, *HADS* Hospital Anxiety and Depression Scale, *PDAS* Pain Disability Assessment Scale

Subsequently, multiple stepwise linear regression analyses was performed to investigate the relationship between PSEQ scores (the response variable) and other variables in female patients (*n* = 112). The explanatory variables included age; pain duration; and NRS, PCS, HADS anxiety, HADS depression, and PDAS scores. We found that PSEQ scores (*y*) were positively correlated with age (*x*_4_) and negatively correlated with HADS depression (*x*_5_) and PDAS (*x*_6_) scores in female patients. These results yielded the following prediction formula: *y* = 32.98 + 0.22*x*_*4*_ − 1.29*x*_*5*_ − 0.32*x*_*6*_ (Table 5).

**Table 5:** Correlations between measured variables and PSEQ scores in female patients

Variable	Partial regression coefficient	Standard error	T-score	*p* value
HADS depression	− 1.29	0.36	− 3.62	0.00
PDAS	− 0.32	0.11	− 2.85	0.00
Age	0.22	0.10	2.24	0.03
Constant term	32.98	6.79	4.86	0.00

*PSEQ* Pain Self-Efficacy Questionnaire, *HADS* Hospital Anxiety and Depression Scale, *PDAS* Pain Disability Assessment Scale

## Discussion

The results of the present study indicated that lower PDAS and lower HADS depression scores correlated with higher PSEQ scores in patients with chronic pain. Neither the site nor the degree of pain correlated with PSEQ scores; however, a longer pain duration was related to higher PSEQ scores in patients with intractable chronic pain. Moreover, advanced age was positively correlated with PSEQ scores in female patients.

In the fear-avoidance model, pain catastrophizing initiates vicious pain spiral and amplifies anxiety and depression, consequently disturbing daily activities, which further exacerbates pain [10–12]. As a protective factor against this vicious pain spiral, confrontation, which was assessed via PSEQ in this study, serves an important role. Previous reports suggested that self-efficacy reflects reciprocity between the self and environment [23], low self-efficacy discourages new undertakings, the continuing lack of mastery experiences may reinforce perceptions of personal inefficacy over time, and high self-efficacy encourages the pursuit of new challenges [14]. In the present study, it was found that the elderly, particularly the female elderly, may learn how to cope with pain in their daily life and exhibit high PSEQ scores. Higher self-efficacy also correlated with diminished pain-related disability in chronic pain patients [15]. The present study also suggested that high self-efficacy correlated with a lower level of pain-related disability. Our previous study reported that the main goal of treatment in the pain liaison outpatient clinic was not pain relief, but rather improved activities of daily living and quality of life, and depression was a risk factor for prolonged pain [24]. In this study, depression was identified as a factor that inhibited self-efficacy.

Our previous report also focused on PCS [25] for assessing pain catastrophizing, which initiates a vicious pain spiral in the fear-avoidance model. In the present study, PSEQ and PCS scores were independent factors in patients with intractable chronic pain, indicating that self-efficacy and pain catastrophizing may coexist independently. We consider that patients with intractable chronic pain should be treated based on the bidirectionality of self-efficacy and pain catastrophizing.

Cognitive behavioral therapy (CBT) is a mode of psychotherapy to treat depression, and multidisciplinary CBT-based treatments have also been introduced in psychosocial fields and chronic pain [26–28]. CBT is considered to increase self-efficacy by providing graded mastery experiences, vicarious learning, and verbal persuasion [17, 29] and improves outcomes in patients with intractable chronic pain. Given these findings, treatments focused on improving the psychological status of patients with depression may enhance self-efficacy and thus, decrease chronic pain impairments.

While it offers some significant benefits to the field, the present study also has some limitations, which warrant discussion. First, patient outcomes were evaluated only at a single time point, including patients in various phases of therapy. There may be an influence of treatment phase on the reported outcomes. Second, given that patients who experienced prolonged pain were usually treated previously at another hospital, pain duration, which predicted self-efficacy here, was inferred. Assessing the exact therapy period and the context of treatment was challenging and thus served as a further, related limitation of the present study. Third, patients who experienced pain for less than 3 months were not included in the present study and were thus not assessed for self-efficacy or pain improvements. We predict that patients with high self-efficacy who experienced a short duration of pain may have experienced more immediate improvements in their pain with any treatment and thus have finished their treatment. Excluding these patients and their clinical courses is a further limitation of the present study. Further prospective study would be needed to prove the relationship between self-efficacy and pain duration.

## Conclusions

This present study demonstrated that higher self-efficacy was correlated with lower pain disability and depression. Further, multidisciplinary therapy, for example, including physical therapy to improve activities of daily living and cognitive behavioral psychological therapy to relieve depression, may improve self-efficacy and thus, result in improved outcomes in patients with intractable chronic pain.

## Data Availability

The datasets analyzed during the current study are available from the corresponding author by reasonable request.

## Abbreviations

CBT: Cognitive behavioral therapy
HADS: Hospital Anxiety and Depression Scale
NRS: Numeric Rating Scale
PCS: Pain Catastrophizing Scale
PDAS: Pain Disability Assessment Scale
PSEQ: Pain Self-Efficacy Questionnaire
